# Variation in tolerance of rice to long-term stagnant flooding that submerges most of the shoot will aid in breeding tolerant cultivars

**DOI:** 10.1093/aobpla/plu055

**Published:** 2014-09-08

**Authors:** Georgina V. Vergara, Yudhistira Nugraha, Manuel Q. Esguerra, David J. Mackill, Abdelbagi M. Ismail

**Affiliations:** 1 International Rice Research Institute, DAPO Box 7777, Metro Manila, Philippines; 2 Indonesian Center for Rice Research, Sukamandi, West Java, Indonesia; 3 Mars, Inc., Department of Plant Sciences, University of California, Davis, CA, USA

**Keywords:** Carbohydrates, rice, stagnant flooding, *SUB1*, tolerance mechanisms.

## Abstract

Stagnant flooding (SF) is a major problem in rainfed lowlands where floodwater of 25–50 cm stagnates in the field for most of the season. We aimed to establish a system for phenotyping SF tolerance and identifying tolerant germplasm through screening of landraces. A total of 626 rice accessions were evaluated over 3 years under control conditions and two levels of SF. Floodwater was raised to 20 cm at 25 or 30 days after transplanting (DAT). In one trial, the depth was increased subsequently by 5 cm a week and in another (severe stress), it was increased to 40 cm at 37 DAT and to 50 cm at 42 DAT. In both trials, water depth was maintained at 50–60 cm until maturity. In all cases, no plant was completely submerged. Plant height, elongation rate and yield were measured at maturity. Genotypes best suited to SF showed moderate elongation of 1.3–2.3 cm day^−1^ under SF. In contrast, semi-dwarf and fast-elongating types performed poorly. Subsequent trials using 18 genotypes, including six pairs of near isogenic lines (NILs) with or without *SUB1* showed that all *SUB1* NILs were sensitive to SF. Five of the other six genotypes contained *SUB1* and were SF tolerant, suggesting the possibility of combining tolerances to complete submergence (*SUB1*) and SF. Stem starch and soluble sugar concentrations were similar under control conditions among the 18 genotypes, but starch was depleted by 37 % under SF, with less depletion in tolerant genotypes. *SUB1* NILs contained similar concentrations of starch and sugars under SF. We conclude that survival and yield under SF are dependent on moderate elongation, high tillering, lesser carbohydrate depletion and higher fertility. The tolerant genotypes identified here performed strongly in both wet and dry seasons and will be used to identify tolerance mechanisms and alleles for use in marker-assisted breeding.

## Abstract (precis)

Long-term stagnant flooding (SF, 50 cm water depth) is a major problem in rainfed lowland rice. We established a phenotyping protocol and identified tolerant landraces. Genotypes best suited to SF showed moderate elongation of 1.3–2.3 cm d^−1^ under SF, but semi-dwarf and fast-elongating types are intolerant. Dwarf varieties containing *SUB1* are sensitive, but taller varieties with *SUB1* are tolerant, suggesting the possibility of combining tolerances to complete submergence and SF. Tolerance of SF is dependent on moderate elongation, high tillering, lesser carbohydrate depletion and higher fertility. Tolerant genotypes identified here will be used for mechanistic studies and in breeding.

## Introduction

Floods caused by heavy rain or outflow of rivers result in severe crop losses worldwide. The losses from these floods are expected to be more severe in future due to climate change. Over 13 million (M) hectares of rice area in India, 3M ha in Bangladesh, 5M ha in Indonesia and 1M ha in Thailand are affected annually by monsoon floods, causing substantial crop losses (Mohanty 1987; Widjaya-Adhi 2000). Continuous monsoon rainfall between mid-June and early October brings about flash floods and complete submergence that is often followed by prolonged stagnant flooding (SF) (Datta 1987; Singh *et al.* 2011).

To facilitate breeding of suitable rice genotypes, variations in flooding depth and duration experienced in practical farming have led to the development of a classification of flood-prone rice environments (Khush 1984; Ismail *et al.* 2010). Complete submergence can occur for a short time (<14 days) following heavy rain or overflowing rivers, causing complete inundation. This is referred to as flash flooding. The extent of damage to rice caused by complete submergence is dependent on floodwater characteristics such as temperature, turbidity and depth. These affect various plant processes such as chlorophyll retention, underwater photosynthesis, carbohydrate accumulation, elongation and survival (Das *et al.* 2009). Rice genotypes tolerant of complete submergence retain their chlorophyll and adopt a slow-growth strategy depicted by limited elongation when submerged. This enables plants to maintain sufficient carbohydrate reserves to sustain metabolism during submergence and also to recover once the floodwater recedes (Setter and Laureles 1996; Xu *et al.* 1996; Ella *et al.* 2003; Das *et al.* 2005; Fukao *et al.* 2006; Sarkar *et al.* 2006; Perata and Voesenek 2007). Tolerant genotypes carrying the submergence-tolerance allele *SUB1A-1* in chromosome 9 can endure complete submergence for up to 2 weeks by limiting leaf extension under water (Xu and Mackill 1996; Septiningsih *et al.* 2009; Singh *et al.* 2009). The physiological mechanisms by which *SUB1A* regulates growth have been thoroughly investigated (Jackson 1985; Jackson *et al.* 1987; Ella *et al.* 2003; Fukao and Bailey-Serres 2008; Bailey-Serres *et al.* 2010; Schmitz *et al.* 2013).

Regulation of elongation in plants is mostly mediated by three phytohormones: ethylene, gibberellic acid (GA) and abscisic acid (ABA). The waterlogged environment slows gas diffusion, thereby entrapping endogenously produced ethylene, resulting in an accumulation of the gas in rice to growth-active concentrations (Satler and Kende 1985). Increased ethylene decreases ABA levels and promotes internodal elongation (Kende *et al.* 1998). Under submergence, *SUB1A* limits ethylene-activated elongation growth by two processes: (i) augmenting GA repressors SLR1 and SLR2, limiting GA responsiveness (Fukao and Bailey-Serres 2008; Bailey-Serres *et al.* 2010), and (ii) enhancing GA catabolism by differentially regulating the genes associated with brassinosteroid synthesis, inducing a GA catabolic gene (Schmitz *et al.* 2013). Both processes limit GA-induced growth, thus conserving carbohydrates for maintenance metabolism and recovery.

Using marker-assisted backcrossing, the *SUB1* locus from the Indian landrace FR13A has been introgressed into several popular varieties, and subsequent evaluation of Sub1 lines showed no differences in grain type or quality from the recurrent parents when grown under control conditions (Siangliw *et al.* 2003; Neeraja *et al.* 2007; Septiningsih *et al.* 2009; Singh *et al.* 2009; Iftekharuddaula *et al.* 2011). Trials in farmers' fields across South and Southeast Asia demonstrated a yield advantage of 1–3 t ha^−1^ in flood-prone areas (Sarkar *et al.* 2006; Singh *et al.* 2009; Vergara *et al.* 2009). A rapid and widespread adoption of Sub1 varieties was observed between 2009 and 2013 in South and Southeast Asia (Mackill *et al.* 2012; Dar *et al.* 2013; Ismail *et al.* 2013; Singh *et al.* 2013).

In deepwater areas, flooding of >100 cm deep can occur for several months. Adapted genotypes accelerate their growth to keep pace with the rising floodwater to escape complete submergence, and this is associated with high carbohydrate consumption. This ‘escape strategy’ is effective when floodwater rises gradually and persists in the field for periods longer than 3 weeks. It can also be effective where the vigour of underwater elongation is sufficient to retain the upper leaves above the water level over periods of up to 37 days (Sakagami *et al.* 2009). Deepwater genotypes can elongate at rates of up to 25 cm day^−1^ (Vergara *et al.* 1976) and can attain a height of 5 m while keeping their top leaves and panicles above the water surface (Jackson and Ram 2003). Recently, knowledge of the physiological mechanisms of rice for tolerance of various flood types was reviewed by Colmer *et al.* (2014). Studies regarding interacting roles of the hormones, ethylene, ABA and GA, and molecular mechanisms of ACC synthase genes, expansins, xyloglucan endo-transglycosylases and other transcriptional regulators involved in shoot elongation of rice under water were reviewed by Vriezen *et al.* (2003). Two genes, *SNORKEL1* (*SK1)* and *SNORKEL2* (*SK2*), involved in the elongation of deepwater rice have recently been identified. Both are transcription factors and have been cloned from the deepwater rice genotype C9285. They seem to function in a way that is contrary to *SUB1A* (Hattori *et al.* 2009). Two other quantitative trait loci (QTLs), *qTIL2* and *qTIL4,* regulate early internode elongation and function in coordination with three major QTLs under deepwater conditions (Nagai *et al.* 2012). These QTLs facilitate significant elongation of internodes and leaves by stimulating GA responsiveness.

Stagnant flooding (prolonged partial flooding; medium deep) occurs in areas when floodwater of 25–50 cm stagnates in the field from a few weeks to several months (Khush 1984; Singh *et al.* 2011). Despite the drastic reduction in survival and yield under these conditions, limited knowledge is available on how rice may survive SF and still produce a crop. In some ways, partially flooded conditions may be similar to deepwater conditions, although germplasm requirements and adaptational mechanisms are different. Tall, fast-elongating deepwater rice varieties perform poorly under SF conditions because of lodging and low fertility. So far, there have been few studies investigating rice tolerance of SF and these are mostly field trials. In trials involving 577 genotypes in West Bengal, India, genotypic differences were observed in survival and grain yield; however, conventional *SUB1A* type submergence-tolerant cultivars such as FR13A and FR43B did not perform well under SF (Datta 1987; Sakagami *et al.* 2009). This shows that submergence-tolerant varieties may not necessarily be suitable in areas affected by SF. Amante (1986) evaluated 25 genotypes across multiple seasons and in different environments (normal and SF through flowering), and observed variation in grain yield and other agronomic characters. Mallik *et al.* (1995) observed that lines with moderate elongation had good survival and higher yield. Semi-dwarf varieties like Swarna and IR64 introgressed with the *SUB1* gene from FR13A showed poor survival at SF depths of 25–30 cm following complete submergence (Singh *et al.* 2011). The rainfed lowland breeding programme at the International Rice Research Institute (IRRI) identified some promising breeding lines with tolerance to both submergence and SF (Mackill *et al.* 2010).

Most modern rice cultivars are not well-adapted to SF conditions, and farmers still use local landraces with poor grain quality and low yields of ∼0.5–1.5 t ha^−1^. More recently, Nugraha *et al.* (2013) tested paired material with and without the *SUB1* locus by imposing 2 weeks of complete submergence followed by a 25- or 50-cm-deep SF treatment. They observed a significant decline in survival and yield, showing that the requirements for tolerance of complete submergence versus prolonged partial submergence are different. Nevertheless, genotypic variation in SF tolerance exists (Singh *et al.* 2009), with possibilities of developing high-yielding varieties with better tolerance. New sources of SF tolerance with higher survival need to be identified, and traits associated with tolerance need to be understood before successful breeding of tolerant high-yielding genotypes can be achieved.

The present 3-year study aimed to establish an effective phenotyping system for SF tolerance and for identifying an SF-tolerant germplasm through large-scale screening of landraces. The study also began the process of identifying major morphological and physiological traits associated with tolerance. The germplasm identified in this study will be useful for future genetic and physiological studies intended to unravel the specific determinants of adaptation to SF conditions and to aid the selection of tolerant lines during breeding.

## Methods

### Field experiments

Between 2007 and 2009, 626 rice genotypes were screened under SF at the IRRI, Los Baños, Philippines. The entries consisted of landraces selected from the Genetic Resources Centre of IRRI based on their origin from flood-prone and deepwater areas, and from selected landraces from South Bangladesh (courtesy of A. Rahman) and advanced breeding lines from the IRRI breeding programme. Three treatments were used: (i) control with shallow flooding of ∼5 cm, (ii) gradual flooding starting at 30days after transplanting (DAT) with 20-cm water depth and increased weekly by 5 cm up to 50–60 cm and (iii) severe flooding at 30 DAT with 20-cm water depth then increased to 40 cm at 37 DAT and to 50–60 cm at 42 DAT. For all treatments, once SF of 50–60 cm was reached, it was maintained through to maturity. In all cases, none of the entries were completely submerged. The land was prepared by plowing and harrowing, and recommended doses of fertilizers (40 : 40 : 40 : 2 N–P–K–Znkg ha^−1^) were incorporated during the second harrowing prior to field levelling. A molluscicide was applied 1 day prior to transplanting to control golden apple snail. Seedlings (21-day-old) were transplanted using one plant/hill with 20 × 20 cm spacing in a randomized complete block design with three replicates, in both control (shallow-flooded) and SF field plots. In 2007 and 2008, seedlings were transplanted in 1 × 1.5 m plots and in 2009, plots of 2 × 3 m were used. The SF treatment started at 30 DAT in the dry season (DS) and at 25 DAT in the wet season (WS). During 2009, 32 genotypes comprising tolerant landraces identified from previous trials, pairs of popular varieties with and without the *SUB1 QTL*, and five advanced breeding lines earlier identified as tolerant of SF, were included. The number of entries used in each trial, flooding conditions and number of best genotypes and survival ranges are summarized in Table 1. The total number of entries evaluated in 2007 and 2008 was 681, including 55 entries selected in 2007 and re-evaluated in 2008.

**Table 1.** Stagnant flooding conditions, number of entries, number of best genotypes and their percentage survival range during the 2007–09 trials at IRRI, Los Baños, Philippines. Gradual flooding started at 30 DAT with a 20-cm water depth and a weekly increase of 5 cm until reaching 50–60 cm. Severe flooding started at 30 DAT with a 20-cm water depth, followed by 40 cm at 37 DAT and 50–60 cm at 42 DAT. Water depth was then maintained through maturity in all treatments. DS, dry season (January to May); WS, wet season (July to November).

Year and season		SF treatment	No. of landraces	No. of breeding lines	No. of checks	Total no. of entries	No. of best genotypes and survival range (%)
2007	DS	Gradual	204	26	2	232	55 (90–100)
	WS	Severe	133	88	2	223	25 (61–89)
2008	DS	Severe	86	26	2	114	10 (40–66)
	WS	Severe	84	26	2	112	10 (51–73)
2009	DS	Severe	15	15	2	32	17 (64–100)
	WS	Severe	15	15	2	32	17 (55–96)

#### Carbohydrate analysis

In the third trial in 2009, seedlings (14-day-old) of 18 genotypes were grown in another field plot and destructively sampled to determine tissue concentrations of starch and soluble sugars. Stagnant flooding treatment was started earlier in this trial, at 22 DAT with a water depth of 20 cm, and increased to 40 cm after 3 days, then to 50 cm after a week. Samples were harvested at 21 DAT, 1 day before the start of the SF treatment, when the water depth was at 5 cm. Samples were then harvested at 36 DAT during the dry and wet seasons of 2009. Fresh samples (stem of the primary tiller) were frozen in liquid nitrogen, freeze-dried and weighed; then 200 mg of ground sample was extracted with 80 % ethanol (v/v) and analysed for carbohydrate concentrations with anthrone (Fales 1951). Starch analysis followed the procedures described in Setter *et al.* (1989). Starch was solubilized in boiling water for 3 h with further hydrolysis using amyloglucosidase and subsequently analysed for free sugars using glucose oxidase (Kunst *et al.* 1988).

#### Data collection and statistical analysis

Data were taken from both control and flooded plots. Plant survival was determined at 50 DAT as the percentage of the remaining hills relative to the total number of hills before the start of the flooding treatment. All data were recorded from inner rows after leaving two hills as borders at both ends of each row. In 2007, plant height was measured weekly from a subset of 50 genotypes using four plants per replicate over 8 weeks. Survival and tiller numbers were recorded for all entries in 2007 and 2008. In 2009, plant height, tiller and panicle number, flowering time and maturity, and grain yield were determined for 17 tolerant genotypes and 2 sensitive checks following IRRI standard procedures (Gomez 1972). Culm width was measured from the base of the stem using a caliper. Statistical analysis was performed using CropStat for Windows version 7.2 (IRRI 2007) for standard analyses of variance and separation of main effects and interaction means. Association between traits was assessed through simple correlation analyses using Excel software.

## Results

### Phenotyping for SF tolerance

During the 2007 DS, flooding was gradually increased by 5 cm each week to a final depth of 50–60 cm after 8 weeks, and then maintained through to maturity. With this gradual rise in water depth, more genotypes showed higher survival than in subsequent trials where the water head was increased to 50–60 cm within 2 weeks (Table 1). Out of 232 entries evaluated in 2007, 55 genotypes showed higher survival in the range of 90–100 % under SF, while the remaining entries had lower, or even no, survivors. Genotypes with high survival rates also produced proportionally more tillers under SF than the sensitive types, ranging from 9 to 20 tillers per hill. This is similar to the numbers produced under control conditions. Generally, sensitive genotypes produced fewer tillers and fewer green leaves that tended to senesce early in association with poor survival and partial lodging of surviving plants. Based on the results of the trials conducted between 2007 and 2009, 16 landraces with survival between 53 and 91 % and three breeding lines with survival of 64–75 % were selected as tolerant, with a mean survival of 75 % compared with 20 % for the SF-sensitive checks (Table 2). This tolerant set reflects considerable variability in numerous agronomic traits including plant height, tillering ability, days to maturity and grain yield when grown under control conditions (Table 3) or in response to SF. The tolerant set of landraces will be useful for future studies of traits associated with SF tolerance and for use in breeding.

**Table 2.** Survival, country of origin and trial periods of promising landraces and breeding lines tolerant of SF (50–60 cm depth) conditions, selected from over 600 entries screened between 2007 and 2009 at IRRI, Philippines. Values are means of the trials listed. IRGC, International Rice Genetics Resources Centre.

Entries	IRGC No.	Country of origin	Survival (%)	Season, trial year
Landraces				
Sossoka Ami 2043	66478	Guinea-Bissau	91.0	DS, WS 2009
Rajasail	8339	Bangladesh	89.5	DS, WS 2009
Patnai 23	25913	Bangladesh	84.0	WS 2008; DS, WS 2009
Sai Bua	40242	Thailand	83.5	DS, WS 2009
Moyna Moti	66818	Bangladesh	81.0	DS, WS 2009
Chiknal	25248	Bangladesh	79.7	WS 2008; DS, WS 2009
Madhukar Code No. NC220	46273	India	79.5	DS, WS 2009
Tojuma	27485	Indonesia	79.0	DS, WS 2009
Dholamon 64–3	6596	Bangladesh	79.0	DS, WS 2009
Sirambe Putih	73842	Indonesia	78.4	WS 2007, DS, WS 2009
Capsule	none	Bangladesh	74.0	DS, WS 2009
Khao Dawk Mali 105	27748	Thailand	70.0	DS, WS 2009
Tilakachari	72965	India	68.9	WS 2007, WS 2008
Ghijoj	114603	Bangladesh	63.4	WS 2007, WS 2009
Jaladhi 1	45858	India	53.2	WS 2007, WS 2008
Breeding lines				
IRRI119		Philippines	71.5	DS, WS 2009
IR70213-9-CPA-AS-UBN-2-1-3-1		Philippines	69.2	DS, WS 2009
IR70181-5-PMI-3-2-B4-1		Philippines	64.1	DS, WS 2009
Means (tolerant)			75.5	
Swarna-intolerant check	117278	India	22.0	2007 2008 2009
IR42-intolerant check	IRRI variety	Philippines	18.0	2007 2008 2009
Means (sensitive)			20.0	
LSD_0.05_			27.4

**Table 3.** Performance of tolerant genotypes and checks under control (C) and SF through maturity and respective percentage differences (% diff) between treatments. Data are means of two trials with three replicates in each trial in 2009 at IRRI, Philippines.^§^Photoperiod sensitive; ^§§^Sossoka Ami 2043 and Tojuma excluded; *, **significant at *P* < 0.05, *P* < 0.01, respectively.

Genotypes (G)	Tillers (no.)			Panicles (no.)			Culm width (mm)			Height (cm)		
	C	SF	% diff	C	SF	% diff	C	SF	% diff	C	SF	% diff
Sossoka Ami 2043^§^	13	12	−6	8	6	−25	6.2	7.8	26.6	156.0	197.5	26.6
Rajasail	14	14	0	14	10	−29	4.7	6.2	31.3	157.3	169.5	7.7
Patnai 23	16	11	−31	12	10	−17	5.9	8.0	35.5	161.6	183.0	13.3
Sai Bua	10	8	−20	10	7	−30	5.9	7.5	27.2	109.6	125.0	14.1
Moyna Moti	14	10	−33	11	10	−9	5.7	6.8	20.1	141.3	159.5	12.9
Chiknal	13	9	−29	11	9	−18	4.3	5.5	27.2	129.6	149.5	15.3
Madhukar Code No. NC220	9	9	0	7	7	0	6.3	8.7	38.4	147.5	195.0	32.2
Tojuma^§^	9	9	0	7	5	−29	6.2	9.8	57.5	157.0	174.0	10.8
Dholamon 64-3	9	9	0	8	8	0	6.0	6.8	13.2	178.8	199.0	11.3
Sirambe Putih	10	9	−10	10	7	−30	6.5	7.8	19.3	170.4	186.0	9.1
Capsule	12	9	−26	12	8	−33	6.2	6.8	10.0	145.2	158.0	8.8
Khao Dawk Mali 105	14	8	−45	12	8	−33	5.5	7.3	33.3	143.2	160.5	12.1
IRRI119	11	10	−8	11	8	−38	4.5	7.0	55.6	115.5	137.0	18.7
IR70213-9-CPA-AS-UBN-2-1-3	11	11	0	10	10	0	5.0	7.0	40.0	110.3	140.0	27.0
IR70181-5-PMI-3-2-B4-1	12	10	−18	11	8	−38	4.8	7.0	45.8	105.9	142.0	34.1
Swarna (intolerant check)	14	8	−36	13	6	−54	5.0	6.0	20.0	96.4	119.0	23.4
IR42 (intolerant check)	20	6	−70	16	6	−63	5.0	6.0	20.0	96.7	115.0	18.9
Means	12	10		11	8		6.0	7.0		136.6	159.4	
LSD_0.05_ (genotype, *G*)	4.36	2.12		3.16	2.78			1.19		39.19	26.50	
LSD_0.05_ (treatment, *T*)		6.19			5.35			0.35			42.95	
LSD_0.05_ (*G*× *T*)		6.34			4.18			1.40			35.80	
Correlation with yield under SF^§§^	0.40			0.18			0.67**			0.70**		
**Genotypes (G)**	**Maturity**			**% Fertility**			**Yield (t ha^−1^)**					
	**C**	**SF**	**% diff**	**C**	**SF**	**% diff**	**C**	**SF**	**% diff**			
Sossoka Ami 2043^§^	170	170	0.0	61.1	53	−13.6	2.0	1.3	−34.0			
Rajasail	125	129	3.2	80.5	71	−12.2	5.2	3.3	−36.5			
Patnai 23	138	138	0.0	61.1	54	−11.7	5.2	3.2	−39.3			
Sai Bua	124	126	−1.6	79.3	63	−20.2	4.8	4.1	−13.3			
Moyna Moti	128	116	−6.3	79.6	64	−19.7	4.3	2.3	−45.8			
Chiknal	108	114	4.2	78.9	87	10.0	4.4	2.1	−52.6			
Madhukar Code No. NC220	128	130	1.6	92.1	81	−12.5	4.7	4.6	−0.8			
Tojuma^§^	174	174	0.0	61.1	31	−48.9	2.4	0.8	−66.3			
Dholamon 64-3	134	135	0.7	73.1	68	−7.5	5.2	4.3	−17.4			
Sirambe Putih	148	155	4.7	84.7	57	−32.6	3.9	3.8	−3.8			
Capsule	125	126	0.8	75.4	57	−25.0	3.5	1.7	−52.3			
Khao Dawk Mali 105	116	114	−1.7	85.4	66	−23.0	2.5	2.0	−19.4			
IRRI119	121	114	−5.8	83.7	66	−21.2	4.2	2.5	−40.5			
IR70213-9-CPA-AS-UBN-2-1-3	115	115	0.0	78.5	76	−3.2	4.0	2.4	−40.0			
IR70181-5-PMI-3-2-B4-1	116	115	−0.9	78.9	76	−3.7	3.8	2.7	−28.9			
Swarna (intolerant check)	111	115	3.6	82.9	18	−78.3	3.9	0.9	−78.2			
IR42 (intolerant check)	118	125	5.9	78.8	18	−77.2	4.0	0.6	−85.0			
Means	130	130		76	52		4.0	2.6				
LSD_0.05_ (genotype, *G*)					17.18			1.23				
LSD_0.05_ (treatment, *T*)					31.29			0.49				
LSD_0.05_ (*G*× *T*)					31.80			2.27				
Correlation with yield under SF^§§^				0.60*								

#### Plant height and elongation ability

**Figure 1.**
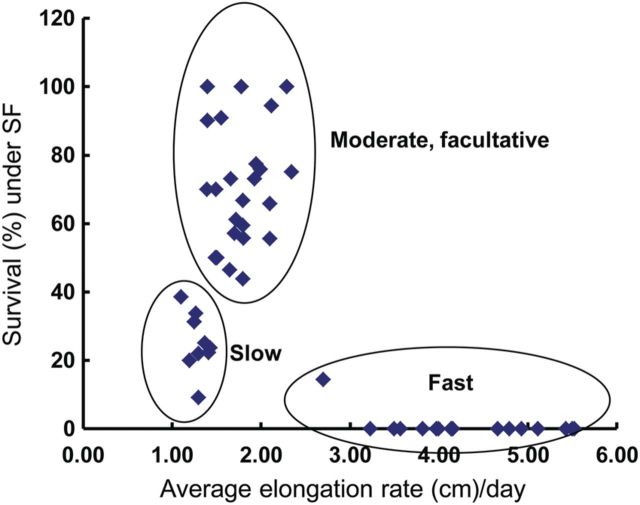
Relationship of plant survival with elongation rate under SF conditions. Data are from 2007 DS. Plant height was measured for 50 genotypes using four plants per genotype for 8 weeks with initial flooding at 20 cm at 30 DAT and increased by 5 cm weekly until reaching 60 cm, then kept through maturity. Survival was counted at 50 days after the start of the SF treatment.

Under SF conditions, substantial differences in plant height were observed. The genotypes showed varying degrees of elongation induced by flooding, demonstrating considerable phenotype plasticity in this trait. From a subset of 50 entries in 2007, plant height ranged between 40 and 80 cm at 7 days of flood; 58–120 cm at 22 days; 84–170 cm at 35 day; and 105–230 cm at 60 days of SF flooding. Genotypic variation in elongation ability under SF was also evident when compared with their counterparts grown under control conditions in three distinct categories (Fig. 1). The first category comprised mostly deepwater landraces wherein SF caused them to elongate by 40–50 % over their height under control conditions, reaching 190–230 cm. Cultivars Kalagyi from Myanmar and ARC12037 from India are examples of lines showing this response. However, most of these tall genotypes lodged severely in standing water thus compromising their survival and final yield. The second category of genotypes comprised semi-dwarf types such as Swarna, a popular variety from India, and IR42. Both were used as sensitive checks. These genotypes elongated by 18–23 % in SF, reaching a height of 105–117 cm upon maturity. They showed poor survival under SF. The third category comprised genotypes showing moderate elongation rates of 10–34 % compared with control conditions, reaching 137–197 cm under SF. Examples of these accessions are Sai Bua and Khao Dawk Mali from Thailand and Rajasail and Patnai 23 from Bangladesh. Lines in this category had the highest survival under SF. Apparently, accessions that elongate especially slowly or quickly survived poorly under SF, while accessions with relatively slower and gradual elongation rates were more tolerant. The results indicate that survival is favoured by an elongation rate of 1.3–2.3 cm day^−1^, which is just sufficient to adjust to an increase in water level.

#### Tillering ability

Variation in tiller production and growth first became visible after 2 weeks of SF. Generally, SF suppressed tillering. However, tiller number was slightly higher, by one to two tillers, during the DS when compared with the wet season across years. Substantial reductions in tiller number were observed in the intolerant variety IR42, which produces only 7–8 tillers under SF compared with 25–38 tillers per hill under control conditions. From a subset of 54 genotypes from 21 countries assessed during the 2007 DS, 39 % had ≤10 tillers, 46 % had 11–15 tillers, 9 % had 16–20 tillers and only 6 % had over 20 tillers under SF **[see Supporting Information]**. In the second trial, conducted during the wet season of 2007, SF treatment was more severe, with faster increase in water depth. In this trial, 223 entries were screened and only 25 accessions showed relatively high survival in the range of 61–89 %. Out of the 25 genotypes, six accessions also produced more tillers under SF compared with other genotypes **[see Supporting Information]**.

During 2008, a total of 114 accessions were phenotyped during the wet season and 112 accessions during the DS, both under severe SF. Plant survival was generally low, with the highest percentage survival of 66 and 73 % for 2008 DS and wet season trials, respectively. Only 10 accessions from both seasons showed higher tiller number (9–15 tillers). The most tolerant landraces were Chiknal, Tilakachari, Jaladhi and Patnai 23 **[see Supporting Information]**.

#### Agronomic traits

In 2009, severe flooding conditions were applied to evaluate the performance of 32 genotypes under SF. These included 13 landraces (Sossoka Ami 2043, Rajasail, Patnai23, Sai Bua, Moyna Moti, Chiknal, Madhukar Code no. NC220, Tojuma, Dholamon 64-3, Sirambeh Puti, Capsule, Khao Dawk Mali 105 and Tilakachari) selected based on survival and tiller number from four previous trials, together with five breeding lines with relatively high SF tolerance (IR70213-9-CPA-AS-UBN-2-1-2, IRRI119, IR70181-5-PMI-3-2-B4-1, IR70181-32 and IR67440-M) and six pairs of NILs (Swarna, Sambha Mahsuri, IR64, BR11, CR1009 and TDK1) with and without *SUB1*, plus IR49830-7 (Sub1) and IR42. These pairs of NILs had been evaluated previously under submergence followed by SF of ∼25 cm by Nugraha *et al.* (2013). During the DS, >64 % of the plants of 17 of the 32 entries survived with 100 % survival in one accession (Tojuma). The mean percentage survival across all tolerant genotypes was 87 %. During the wet season, the same entries were re-evaluated with flooding applied 5 days earlier. The same 17 tolerant entries showed over 74 % survival, with the highest being 96 % (Patnai 23). Responses of these genotypes and the intolerant checks, Swarna and IR42 under control and SF conditions across the two seasons are summarized in Table 3. The mean percentage survival of the tolerant genotypes was 75 % and that of the sensitive genotypes was only 20 %. Effects of genotype and genotype × treatment were significant, but that of genotype × season was not significant, indicating that the rank of these genotypes is consistent across seasons.

Severe SF resulted in poor panicle development, higher spikelet sterility, less grain filling, and generally poor survival and yield. Promising genotypes maintained 9–14 tillers and showed higher survival and spikelet fertility than the intolerant checks (Table 3). Correlation of tiller number with survival was positive and significant (*r* = 0.54***). Stagnant flooding reduced tillering by as much as 70 % in intolerant IR42, but was maintained or slightly reduced (0–10 % reduction) in six tolerant landraces and the two genotypes, IRRI119 and IR70213-9-CPA-AS-UBN-2-1-3-1 (Table 3). Plant height was significantly different among genotypes under both control and SF conditions. Generally, genotypes that are taller under control conditions and with the ability to elongate partially under SF are more tolerant than shorter genotypes. From the mean data of tolerant genotypes under control conditions, we estimated that an ideal plant height for candidates for SF tolerance (∼50–60 cm) would be in the range of 130–140 cm, and have the ability to elongate further under SF to reach 160–170 cm.

Panicle number and development are severely affected by prolonged flooding. Under severe SF, 4 out of the 15 tolerant entries in Table 3 (Rajasail, Patnai 23, Moyna Moti and IR70213-9-CPA-AS-UBN-2-1-3-1) produced at least 10 panicles while Tojuma only had 5 panicles. Tojuma is photoperiod-sensitive, which delayed its maturity. Under SF, the sensitive checks Swarna and IR42 produced six panicles each and showed the highest relative reductions in panicle number (54 and 63 %, respectively), compared with control conditions.

No significant differences between lines were observed in culm thickness under control conditions; however, culms became thicker under SF where stem diameter (shoot base) increased by a range of 10–45 % over those in nonflooded conditions (Table 3), and genotypes varied significantly in the extent of SF-induced culm thickening. Mean culm thickness of the intolerant genotypes Swarna and IR42 was 6.0 mm, while 14 out of the 15 tolerant genotypes (Table 3) had thicker culms. Visual comparison of culms between control and SF conditions showed that the increase in culm thickness also increased hollowness, which might aid further in root aeration. However, no aerenchyma measurements were taken to compare tolerant and sensitive genotypes. The correlation between culm thickness and yield is positive and significant (*r* = 0.67**).

Stagnant flooding delayed maturity in some genotypes by about 2–4 days, as seen in Rajasail, Sai Bua and Chiknal. However, three genotypes (Khao Dawk Mali, IRRI119 and IR42) matured earlier in SF, while others showed no changes in maturity duration under SF. Tolerant genotypes showed a wide range of maturity durations, ranging from 108 to 170 days under control conditions and from 114 to 170 days under SF conditions. This range will be useful in selecting donor parents based on the desired maturity class in new breeding lines designed for different latitudes. Six of the 15 tolerant genotypes matured in <120 days under SF, including the two elite breeding lines (Table 3). Two genotypes, Sossoka Ami 2043 and Tojuma, are photoperiod sensitive and thus, matured late (170 days). Their maturity duration was not affected by SF treatment.

Spikelet fertility was reduced by SF by as much as 78 % in intolerant checks. Percentage fertility correlated positively with yield under SF (*r* = 0.60*; Table 3). Unfilled grains mostly occurred at the base of the panicle under SF. Tolerant genotypes with over 70 % fertility include Rajasail, Chiknal, Madhukar, IR70213-9-CPA-AS-UBN-2-1-3-1 and IR70181-5-PMI-3-2-B4-1. These genotypes had grain yields ranging from 2.1 to 4.6 t ha^−1^ under SF. Stagnant flooding also significantly reduced grain yield. The mean yield under control conditions was 4 t ha^−1^, but decreased to 2.6 t ha^−1^ under SF across genotypes. Grain yield of the intolerant checks Swarna and IR42 declined by 78 and 85 %, respectively, under SF. In contrast, some tolerant genotypes such as Madhukar Code NC220 and Sirambe Putih showed no significant decline in yield under SF. Madhukar Code NC220 had the highest yield under SF (4.64 t ha^−1^) followed by Dholamon 64-3 (4.32 t ha^−1^) and Sai Bua (4.14 t ha^−1^). However, two of the tolerant breeding lines that showed high survival under SF had relatively low yields (2.4–2.7 t ha^−1^), suggesting that selection for survival alone under SF will not necessarily improve productivity.

### Carbohydrate analysis

**Figure 2.**
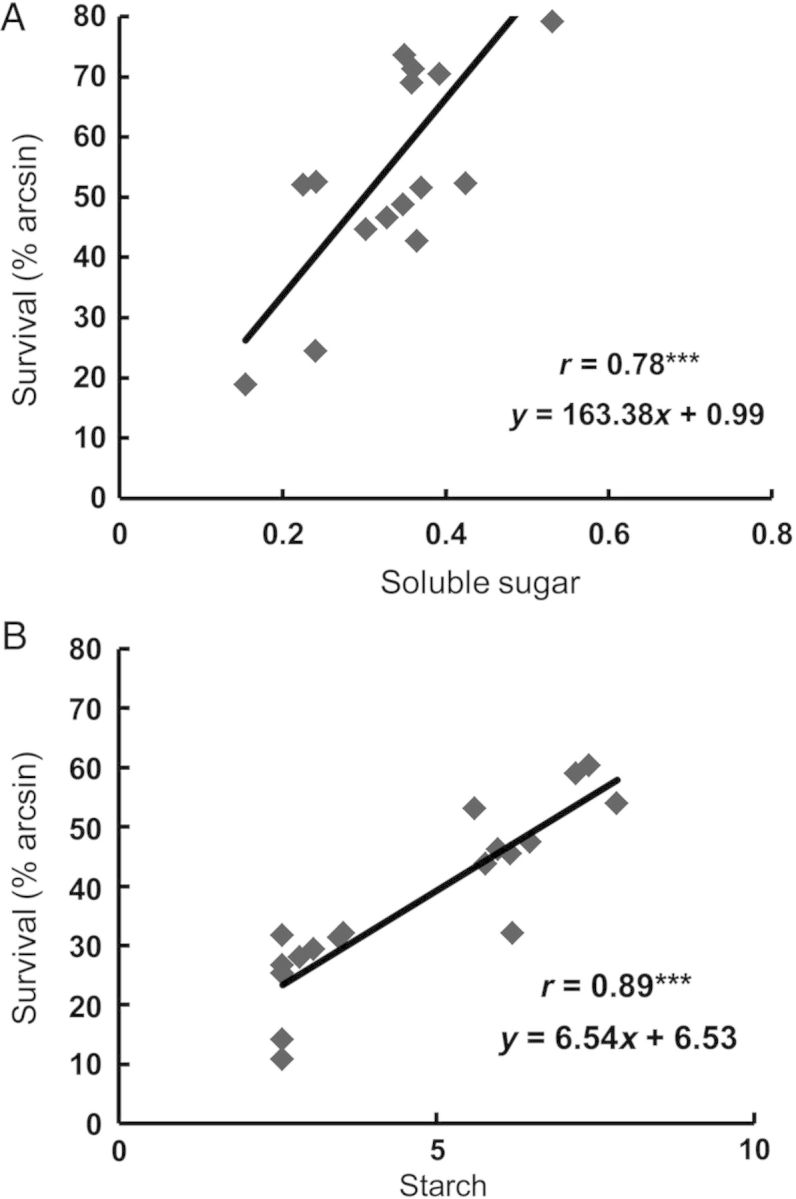
Correlation between plant survival under SF and percentage concentration (dry weight basis) of soluble sugars (A) and starch (B). Data are from 18 rice genotypes evaluated during 2009 DS after 2 weeks of SF maintained at a depth of 50 cm.

We also investigated changes in soluble carbohydrate concentration during SF in tolerant and sensitive genotypes. We evaluated pairs of near isogenic lines (NILs) with and without *SUB1* to test whether the presence of this gene offers benefits in terms of carbohydrate conservation after 2 weeks of SF. Genotypes tolerant of SF but with or without *SUB1* were also included in this evaluation. Soluble sugars and starch were quantified in stems of primary tillers. Under control conditions, soluble sugar concentration did not differ significantly between genotypes at 21 DAT (before treatment; Table 4). After 15 days of SF stress, differences between control and SF conditions were significant, and genotypes showed reductions in soluble sugar concentrations under SF. Genotype × treatment effect was also significant during both dry and wet seasons. The mean performance of the subset of Sub1 introgression lines from two season trials showed that these did not differ significantly from their respective recurrent parents in stem soluble sugar concentrations **[see Supporting Information]**. A greater loss in stem soluble sugars was noted in the sensitive genotypes, decreasing by 45 % under SF. In contrast, tolerant genotypes showed only a 35 % depletion compared with control conditions. The SF-tolerant genotype IRRI119, which also contains the *SUB1* locus, had consistently higher concentrations of soluble sugars than the intolerant lines during both dry and wet seasons. At 36 DAT (15 days under SF), the highest concentration of soluble sugars was observed in IR67440-M in the DS (0.53 %) and in IRRI119 (0.95 %) in the wet season of 2009. Both these genotypes showed higher tolerance to SF. A strong positive association was observed between plant survival and concentration of soluble sugars in the stem following SF (*r* = 0.78** during the 2009 DS (Fig. 2A) and *r* = 0.77** during the 2009 wet season; figure not shown). The variance for soluble sugars at 36 DAT (15 days SF) was not homogeneous between the two seasons in our trials.

**Table 4.** Soluble sugar concentration (% of dry weight) at 21 and 36 DAT and following 2 weeks SF of rice genotypes with (+) and without (–) the *SUB1* locus during 2009 DS and WS. **significant at *P*< 0.01, n.s., not significant.

Genotypes (G)	*SUB1*	SF tolerance	DS2009			WS2009		
			Control	Control	SF	Control	Control	SF
			21 DAT	36 DAT	36 DAT	21 DAT	36 DAT	36 DAT
IR64-Sub1	+	Intolerant	0.52	0.75	0.30	0.66	0.72	0.48
IR64	−	Intolerant	0.51	0.87	0.35	0.52	0.68	0.49
Swarna-Sub1	+	Intolerant	0.52	0.88	0.23	0.60	0.80	0.55
Swarna	−	Intolerant	0.59	1.01	0.31	0.57	0.78	0.54
Sambha Mahsuri-Sub1	+	Intolerant	0.54	0.94	0.23	0.57	0.75	0.71
Sambha Mahsuri	−	Intolerant	0.45	0.81	0.24	0.61	0.74	0.71
BR11-Sub1	+	Intolerant	0.48	0.83	0.48	0.66	0.89	0.41
BR11	−	Intolerant	0.53	0.77	0.36	0.74	0.82	0.41
CR1009-Sub1	+	Intolerant	0.57	0.93	0.37	0.55	0.85	0.30
CR1009	−	Intolerant	0.49	0.88	0.30	0.74	0.86	0.37
TDK1-Sub1	+	Intolerant	0.57	0.82	0.39	0.61	0.68	0.95
TDK1	−	Intolerant	0.56	0.83	0.41	0.54	0.88	0.87
IR49830-7	+	Intolerant	0.55	1.05	0.33	0.52	0.93	0.42
IR70213-9-CPA-AS-UBN-2-1	+	Tolerant	0.52	0.76	0.36	0.60	0.72	0.87
IRRI119	+	Tolerant	0.49	0.84	0.38	0.54	1.07	0.95
IR70181-5-PMI-3-2-B4-1	+	Tolerant	0.58	1.04	0.49	0.67	0.87	0.42
IR70181-32	+	Tolerant	0.51	1.01	0.49	0.77	1.00	0.73
IR67440-M	−	Tolerant	0.41	0.70	0.53	0.58	0.77	0.60
Means			0.52	0.87	0.37	0.61	0.82	0.61
LSD_0.05_ (*G*)			n.s.	0.30	0.20	n.s.	0.32	0.10
Season (DS vs.WS)**								
Control vs. SF**								
LSD_0.05_ (*G*× SF) = 0.06

All genotypes had similar stem starch concentrations before the start of the SF treatment (21 DAT). However, after 2 weeks of SF, genotype, treatment and genotype × treatment effects were all statistically significant. In contrast to soluble sugars, starch concentrations were statistically similar across seasons. Starch concentration in stems decreased substantially under SF (Table 5). This decline was not significantly different in Sub1 and non-Sub1 NILs, suggesting that *SUB1* had no effect on this trait under SF. The difference in starch concentration between SF-tolerant and intolerant genotypes was, however, considerable. Under SF, stem starch concentration of the sensitive genotypes was depleted by as much as 35–75 % compared with controls in both dry and wet seasons. Starch in the intolerant Sub1 NILs, Swarna and Swarna-Sub1 was highly depleted (by 74 and 66 %, respectively), following SF, whereas the tolerant genotypes IR67440-M and IRRI119 showed low starch depletion (11 and 23 %, respectively), following SF. A strong positive association was observed between plant survival and stem starch concentration under SF in the DS (*r* = 0.89**, Fig. 2B) and the wet season (*r* = 0.79**) of 2009.

**Table 5.** Stem starch concentration (% of dry weight) at 21 and 36 DAT and following 2 weeks SF of rice genotypes with (+) and without (−) the *SUB1* locus during 2009 DS and WS. ***significant at *P* < 0.001, n.s., not significant.

Genotypes (G)	*SUB1*		DS2009			WS2009		
		SF	Control	Control	SF	Control	Control	SF
		Tolerance	21 DAT	36 DAT	36 DAT	21 DAT	36 DAT	36 DAT
IR64-Sub1	+	Intolerant	5.29	8.10	2.60	5.08	7.80	3.70
IR64	−	Intolerant	4.43	6.60	6.50	4.33	6.80	4.80
Swarna-Sub1	+	Intolerant	4.56	7.40	0.70	5.44	9.60	3.80
Swarna	−	Intolerant	4.90	6.50	2.50	4.95	9.70	3.00
Sambha Mahsuri-Sub1	+	Intolerant	4.71	6.90	3.50	5.45	8.90	5.20
Sambha Mahsuri	−	Intolerant	4.58	6.10	3.50	4.50	8.20	3.60
BR11-Sub1	+	Intolerant	4.55	8.20	3.10	5.12	8.00	3.90
BR11	−	Intolerant	5.09	8.00	6.00	5.74	7.70	5.10
CR1009-Sub1	+	Intolerant	5.58	8.00	2.30	4.56	7.40	4.00
CR1009	−	Intolerant	5.20	7.70	3.50	5.22	6.20	3.90
TDK1-Sub1	+	Intolerant	5.34	6.20	5.20	5.59	9.90	3.80
TDK1	−	Intolerant	4.51	6.70	5.80	5.56	7.70	5.00
IR49830-7	+	Intolerant	4.87	6.80	2.80	4.90	8.70	4.00
IR70213-9-CPA-AS-UBN-2-1	+	Tolerant	5.01	6.60	5.80	5.28	7.10	4.80
IRRI119	+	Tolerant	5.41	7.70	6.40	4.96	8.50	6.10
IR70181-5-PMI-3-2-B4-1	+	Tolerant	4.68	7.90	3.50	5.16	9.30	4.30
IR70181-32	+	Tolerant	5.48	7.80	6.10	5.33	9.90	4.80
IR67440-M	−	Tolerant	5.25	6.20	5.80	5.65	6.90	5.90
Means			4.97	7.19	4.20	5.15	8.30	5.70
LSD_0.05_ (*G*)			n.s.	1.70	2.70	n.s.	1.00	1.20
Season (DS vs.WS)^ns^								
Control vs. SF***								
LSD_0.05_ (*G*× SF) = 0.36								

## Discussion

Plants have developed numerous adaptive responses that allow them to survive different types of floods and oxygen impedance (Insausti *et al.* 2001; Liao and Lin 2001; Colmer and Voesenek 2009; Colmer *et al.* 2014). Rice generally can tolerate flooded soil when flooding is relatively shallow (5–20 cm), as recently reviewed by Kirk *et al.* (2014). However, few landraces can tolerate transient complete submergence experienced in flash-flood areas or in longer-term partial floods as in stagnant flood and deepwater areas. These landraces are usually identified from affected areas after phenotyping large sets of germplasm. Characterization of flooding stress, including depth, duration and characteristics of the floodwater (Das *et al.* 2009), together with the development of effective phenotyping protocols that simulate the stress at target sites, is essential if tolerant donors are to be correctly identified. Such characterization will also assist in determining the physiological and genetic determinants of tolerance and in breeding programmes. A classic example is the progress made with the *SUB1A* (Fukao *et al.* 2006; Xu *et al.* 2006) and *SK1/SK2* (Hattori *et al.* 2009) genes. The *SUB1A* gene had been characterized and its effects evaluated in different genetic backgrounds and subsequently made available to farmers in affected areas (Bailey-Serres *et al.* 2010; Mackill *et al.* 2012, Ismail *et al.* 2013). However, information on SF tolerance in rice is scant even though this stress is causing yield reductions in millions of hectares in Asia (Singh *et al.* 2011). The present study evaluated rice responses to SF (∼50–60 cm) with aims of establishing effective phenotyping protocols, identifying tolerant genotypes and finding readily quantifiable phenotypic indicators of tolerance.

Stagnant flooding causes hypoxia stress in rice, severely impeding its survival and growth (Singh *et al.* 2009; Singh *et al.* 2011; Colmer *et al.* 2014). In the first trial in our study, a diverse set of 204 landraces from 21 countries and 26 breeding lines from IRRI were evaluated for SF tolerance using survival, tillering ability and plant height as descriptors of performance. Survival rate in this trial was high, with 24 % of the entries having 90–100 % survival. The survival of the intolerant checks IR42 and Swarna was relatively low, at 34 and 38 %, respectively. This demonstrated the ability of most rice landraces collected from flood-prone areas to adapt to gradual increases of water depth. However, wide variation in survival, tillering and elongation ability in response to SF among genotypes indicated that sufficient genetic variability is present for exploitation through breeding, and that several largely independent physiological mechanisms probably underpin this variation.

Tiller number generally decreased under SF, with considerable variation among genotypes, ranging from 10 to 90 %. Three groups of genotypes were identified with slow, moderate or fast elongation. Tolerant genotypes elongate moderately, at a rate of 1.3–2.3 cm per day. Fast-growing types, which were less tolerant, could increase the shoot length by as much as 5 cm per day. Moderate elongation under SF increased the ultimate plant height by 12–49 %, resulting in heights of more than twice the depth of the floodwater. This is probably enough to project sufficient leaf area above water for adequate aeration and photosynthesis. Extensive shoot elongation under SF is not conducive to survival as it consumes much energy and results in tall, spindly plants and leaves that usually lodge and disintegrate in the floodwater. This is unlike the case in deeper water areas, where floodwaters provide support for elongating shoots. At the other extreme, the semi-dwarf and slow-elongating types also have poor survival and low tillering in SF, probably because the majority of leaves stay submerged, resulting in poor aeration and assimilate supply that leads to their premature degradation.

In the second screening trial, SF was imposed by abruptly raising the depth of the floodwater from 20 to 50 cm within 2 weeks as a more stringent test of SF. This improved the identification of tolerant lines and better simulated the conditions in the field created by successive heavy rains. Percent survival was generally lower than that observed in the first trial, with only 11 % of the 223 entries showing good survival rates of 61–89 %, while survival of the intolerant checks was only 10 %.

During the first four trials, plant survival, elongation and tillering ability under SF were found to be effective in selecting the most tolerant genotypes. Besides, additional traits including panicle number, spikelet fertility, culm width, days to maturity and grain yield were assessed. From the six trials conducted during this study, it became clear that severe stress imposed early during the vegetative stage, i.e. increasing the floodwater depth of 20–30-day-old seedlings to reach 50–60 cm within 2 weeks, followed by maintaining that depth through maturity, was effective in identifying the most tolerant genotypes. This protocol will be used in future to identify tolerant donors, for studying tolerance mechanisms and for evaluating breeding material.

The results of the 2009 trial showed that selection for grain yield under SF could be effective in identifying tolerant genotypes (Table 3) and that several traits are associated with SF tolerance. Of these traits, plant height, culm width and spikelet fertility correlated positively with yield under SF. The few tolerant genotypes identified in this study showed limited elongation and in most of cases, elongation was induced by SF (facultative elongation). These genotypes did not lodge because of the increase in their culm thickness, had higher tiller number and more fertile panicles and consequently produced higher grain yields at final harvest.

Facultative elongation is desirable for tolerance of SF conditions. Partial flooding could cause ethylene entrapment in roots and lower shoots, causing fast underwater extension of the shoot (Musgrave *et al*. 1972; Satler and Kende 1985; Jackson 2008). It has been shown that increased ethylene concentration promotes elongation in rice plants (Kende *et al.* 1998), an escape strategy during flooding. Rice genotypes without the *SUB1A* locus (null allele) or with the *Sub1A-2* (intolerant allele due to single-nucleotide substitution) may double their plant height when subjected to high ethylene or to complete submergence at the vegetative stage, though elongation in plants with the *Sub1A-1* (tolerant allele) is restricted (Xu *et al.* 2006; Singh *et al.* 2010). The feedback control for repressing elongation under transient complete submergence caused by *SUB1A* gene has been well established (Fukao and Bailey-Serres 2008; Schmitz *et al.* 2013). *SUB1A* (an ethylene-response factor, or ERF, in chromosome 9) enhances synthesis of brassinosteroids, which increase the expression of a GA catabolic gene, *GA2ox7*, decreasing GA concentration and subsequently, suppressing elongation (Schmitz *et al.* 2013). In contrast the Snorkel genes, *SK1/SK2* (ERFs in chromosome 12), control rapid elongation in deepwater rice and the expression of these genes is induced by increased ethylene (Hattori *et al.* 2009). The effectiveness of these QTLs under SF needs to be evaluated.

The increase in culm width in tolerant rice genotypes is essential to avoid damaging lodging in standing water. We also observed an increase in aerenchyma formation at the leaf base under SF (data not shown), which could further enhance aeration of roots and flooded shoot parts (Kirk *et al.* 2014). Steffens *et al.* (2011) observed enhanced development of aerenchyma caused by submergence or treatment with the ethylene-releasing compound ethephon in all internodes of the deepwater rice variety Pin Gaew 56 and in two lowland rice varieties, albeit to a lesser degree in the latter. Genotypes subjected to SF in the present study showed variation in the extent of increase in culm width, with the majority of tolerant genotypes developing thicker stems in the range of 8–9 mm, suggesting that this range is probably optimum under SF and, together with increased aerenchyma formation, could play a role in tolerance to SF. Further studies will confirm this association and its role as a selectable trait in breeding.

High tillering ability is an important trait for high yield under SF and was correlated positively with survival and panicle number. The mechanism by which SF suppresses tillering in sensitive genotypes is largely unknown. It could potentially involve the accumulation of ethylene that may then prevent tiller formation and/or trigger early senescence of tiller primordia. Tolerant landraces showed less reduction in tillering ability than intolerant checks during SF. Prolonged flooding also reduced panicle formation by over 50 % in the intolerant genotypes, while most tolerant landraces and breeding lines showed 0–30 % reduction. Tiller and panicle number, however, were less correlated with yield under SF in this study. This may be because of a few tolerant landraces with poor spikelet fertility under SF.

Landraces with high survival and tillering ability under SF have thicker culms and high fertility. These genotypes will be useful as donor parents in breeding improved adaptability and grain yield under prolonged flooding. Six of these landraces, Rajasail, Patnai 23, Sai Bua, Madhukar Code No. NC220, Dholamon 64-3 and Sirembeh Puti, showed reasonably high yields of >3.0 t ha^−1^, with relatively smaller reductions in the grain yield of ∼18 %, compared with an average of 80 % reduction in the grain yield of Swarna and IR42. Crosses can be made to combine the tolerance traits of these landraces in the high-yielding backgrounds of modern varieties to improve adaptability and enhance grain yield and quality. Furthermore, discovery of major loci or genes associated with each of these traits will facilitate faster progress in breeding, as most of these traits are apparently independent and quantitative.

Three IRRI breeding lines, IRRI119, IR70213-9-CPA-AS-UBN-2-1-3-1 and IR70181-5-PMI-3-2-B4-1, with relatively high tolerance to SF also possess the tolerant *SUB1A* allele, while pairs of NILs with and without the *SUB1A* allele developed in the background of popular varieties were all intolerant. This supports the view that the possession of the *SUB1A* allele is unrelated to adaptation to SF conditions. Singh *et al.* (2011) subjected Swarna and Swarna-Sub1 to 50 cm of SF and observed a decline in survival by 16 and 17 %, whereas the taller breeding line IR49830 (with *SUB1A*) showed superior survival with only a 2 % reduction. Lines with the *SUB1* gene combined with intermediate elongation ability will be more adapted to SF affected areas (Sarkar and Bhattacharjee 2011).

Here we used six pairs of NILS with and without the *SUB1A* allele to provide more reliable evidence for the role of *SUB1* under SF. All 12 genotypes were equally intolerant, again suggesting that survival in SF is independent of *SUB1* but more dependent on the genetic background, particularly plant height and ability to further elongate under SF. The fact that five of the SF-tolerant genotypes (Table 4) have the tolerant *SUB1A* allele provides clear evidence that it is possible to combine both *SUB1* and SF tolerance in a single background, even though the two tolerance mechanisms are opposite (quiescence for *SUB1* and partial elongation for SF). Further studies are needed to understand how these two contrasting mechanisms are regulated. Our results also show no significant delay to maturity in the tolerant genotypes under SF. This is an important feature to retain in future breeding work. Early-maturing, SF-tolerant varieties are desirable in some flood-affected areas in Asia and Africa. Genotypes combining *SUB1* and SF will be useful in fields where complete submergence commonly precedes or succeeds SF any time during the season.

This study also investigated the importance of maintaining high soluble carbohydrates during SF. The essential role of carbohydrate content in tolerance of complete submergence during germination (Ismail *et al.* 2009, 2012) and vegetative stages (Jackson and Ram 2003; Das *et al.* 2005; Xu *et al.* 2006) has been well established. Waterlogging was reported to enhance anaerobic respiration and increase consumption of accumulated carbohydrates, while reducing the photosynthetic rate (Mazaredo and Vergara 1982; Setter *et al.* 1997, Das *et al.* 2005; Nugraha *et al.* 2013). Maintenance of carbohydrate supply during flooding is therefore important to sustain metabolism and growth and to maintain high grain yield as SF usually persists throughout the season. Pre- and/or post-submergence concentrations of non-structural carbohydrates were frequently associated with enhanced survival after complete submergence (Mallik *et al.* 1995; Sarkar *et al.* 1996; Ram *et al.* 2002; Jackson and Ram 2003; Das *et al.* 2005). In the present study, we observed that pre-flooding carbohydrates of rice seedlings at 21 DAT were not significantly different in 18 genotypes and were not associated with SF tolerance. These findings were similar to that of Nugraha *et al.* (2013), wherein carbohydrate concentrations in shoots measured before flooding were unrelated to survival after complete submergence followed by SF treatments. After 15 days of SF we observed significant variations in genotypic responses and treatment effects for both sugar and starch concentrations with significant genotype × treatment interaction. Higher correlations of carbohydrates with survival were more apparent during the DS and may be due to better solar radiation and photosynthesis.

The results generally show that all genotypes had less soluble sugars and starch following 2 weeks of SF, where stem sugars and starch concentrations decreased by over 45 % relative to the control values. However, the tolerant genotypes showed significantly less starch depletion than the intolerant genotypes. Under partial submergence, the translocation of carbohydrates from the stem and elongating internodes is accelerated (Colmer *et al.* 2014). In deepwater rice, 3 days of partial submergence caused a 65 % reduction in stem starch while translocation of ^14^C-labelled sugars in leaves increased by 26-fold (Raskin and Kende 1984). We observed that post-flooding carbohydrates were positively associated with survival. Following complete submergence, Das *et al.* (2005) reported that shoot carbohydrates remaining after submergence were more vital for survival than total non-structural carbohydrates that accumulate before flooding. As is the case with complete submergence, our results suggest that the capacity of the genotypes to maintain higher carbohydrates during partial flooding is also essential for survival and for higher grain yield under this stress.

In this study, we did not observe the benefit of the presence of *SUB1A* in sustaining stem starch accumulation when plants are partially flooded. Six pairs of NILs contrasting in *SUB1A* maintained similar sugar and starch concentrations after 2 weeks of 50-cm SF. Nugraha *et al.* (2013) found significant differences between Sub1 and non-Sub1 lines subjected to complete submergence followed by ∼25 cm of SF. However, no differences in carbohydrates were found when 12 days of complete submergence were followed by ∼50 cm of SF. Interestingly, some Sub1 genotypes like IRRI119 performed consistently well under SF, had the highest concentration of soluble sugars and had less depletion of starch following SF. This genotype produced a mean yield of 2.5 t ha^−1^ under SF, which is 1.5 t ha^−1^ higher than the mean yield of the intolerant varieties. The ability to tolerate SF through moderate elongation with the maintenance of sufficient carbohydrates during SF may have contributed to the good performance of IRRI119. The presence of *SUB1A* would be an advantage when complete submergence precedes or follows SF.

Rice plants accumulate more starch in their culms than in their leaves. Ishimaru *et al.* (2008) showed that maintenance of higher starch may contribute to the rigidity of the upper portion of the culm and may be responsible for higher lodging resistance. Less starch depletion in the tolerant genotypes could thus contribute to both growth and maintenance, as well as to lodging resistance required under medium-deep SF. Under control conditions, higher carbohydrate reserves are also needed before heading to enable transport to the panicles and improve grain filling (Ishimaru *et al.* 2007). During prolonged SF, carbohydrates utilized in elongation may incur a penalty of lower grain yield, as supported by a substantial reduction in harvest index (grain/total shoot weight) and yield as water depth increases (Colmer *et al.* 2014). Whether higher carbohydrate levels in the stems of tolerant genotypes contribute to SF tolerance by physically strengthening their culms, enhancing their elongation growth or providing storage reserves during grain filling or, is solely a consequence of better photosynthetic capacity due to better exposure to solar radiation, awaits further studies.

## Conclusions

Following screening of 626 rice accessions, several landraces and breeding lines were identified as being tolerant to SF. Flooding by imposing a water depth of 20 cm at 30 DAT, then raising the water depth by ∼20 cm in each of two subsequent weeks to reach 50–60 cm and maintaining this depth through grain filling were effective in identifying tolerant rice genotypes. This approach will be used for screening during breeding for improved tolerance to SF. Besides survival and yield, a range of genotypic variations were observed in several traits that are associated with tolerance, including SF-induced moderate shoot elongation, enhanced tillering ability, thickened culms, better fertility and higher non-structural carbohydrates. Apparently, tolerance to SF requires incorporation of most, if not all, of these traits, suggesting a complexity to tolerance. The SF-tolerant accessions identified in this study will be useful as donors in breeding and for further mechanistic studies. Furthermore, characterization of the traits associated with tolerance in these lines will facilitate refining of breeding strategies and help accelerate progress in developing tolerant varieties.

## Sources of Funding

The research work was supported by the Bill & Melinda Gates Foundation through the STRASA (Stress-Tolerant Rice for Africa and South Asia) project.

## Contribution by the Authors

G.V.V., A.M.I. and D.J.M. conceived the study and designed the experiments. G.V.V. carried out the experiment under the supervision of A.M.I. with technical assistance from Y.N. and M.Q.E. G.V.V. and A.M.I. drafted the manuscript. All authors checked and approved the final draft.

## Conflicts of Interest Statement

None declared.

## Supporting Information

The following Supporting Information is available in the online version of this article–

**Table S1.** Performance of a subset of landraces from 21 countries showing variation in survival and tillering under gradual SF starting at 30 days after transplanting with 20 cm depth and increased by 5 cm weekly to a final flooding depth of 60 cm in 2007 DS trial at International Rice Research Institute (IRRI), Philippines. Data for percentage survival are means of three replicates. Data for tiller number are from four plants/replicate × two replicates.

**Table S2.** List of genotypes with best survival and their tiller number under severe stagnant flooded conditions (20 cm flooding at 30 days after transplanting (DAT) followed by 40 cm at 37 DAT and 50–60 cm depth at 42 DAT) through maturity in the 2007 wet season trial (from 223 entries); 2008 DS (from 114 entries); 2008 wet season (from 112 entries) and their percentage survival. Data are from three replicates in each trial.

**Fig. S1.** Comparison of stem starch (% of dry weight) (A) between Sub1 and non-Sub1; (B) between SF-tolerant and intolerant genotypes; and soluble sugars (C) between Sub1 and non-Sub1; and (D) between SF-tolerant and intolerant genotypes. Data are means from 2009 and analysed using Fishers' test at *P*< 0.001, n.s., indicate not significant; *Significant at *P*< 0.05. Data were collected before treatment (21 DAT) and after 15 days under control and SF (36 DAT).

Additional Information
