# Supplementary material for: Variation in tolerance of rice to long-term stagnant flooding that submerges most of the shoot will aid in breeding tolerant cultivars
Source: AoB Plants. 2014 Sep 8;6:plu055. doi: 10.1093/aobpla/plu055 (PMC4196555; doi:10.1093/aobpla/plu055)
Supplement: Additional Information [file supp_6_plu055_index.html]

Variation in tolerance of rice to long-term stagnant flooding that submerges most of the shoot will aid in breeding tolerant cultivars — Additional Information 

# Variation in tolerance of rice to long-term stagnant flooding that submerges most of the shoot will aid in breeding tolerant cultivars

## Additional Information

Additional Information

**Files in this Data Supplement:**

- Supporting Information - docx file
