# Supplementary material for: Variation in tolerance of rice to long-term stagnant flooding that submerges most of the shoot will aid in breeding tolerant cultivars
Source: AoB Plants. 2014 Sep 8;6:plu055. doi: 10.1093/aobpla/plu055 (PMC4196555; doi:10.1093/aobpla/plu055)
Supplement: Additional Information [file supp_plu055_plu055supp.docx]

**Supplemental Table 1.** Performance of a subset of landraces from 21 countries showing variation in survival and tillering under gradual stagnant flooding starting at 30 days after transplanting with 20 cm depth and increased by 5 cm weekly to a final flooding depth of 60 cm in 2007 dry season trial at IRRI, Philippines. Data for percentage survival are means of three replicates. Data for tiller number are from 4 plants/replicate x 2 replicates.

|  |  | Country of | ___% Survival___ | | ___Tiller no.__ | |
| --- | --- | --- | --- | --- | --- | --- |
| IRGC. No | VARIETY NAME | Origin | Control | SF | Control | SF |
| 237 | TKM6 | India | 100.0 | 75.6 | 25 | 12 |
| 650 | MTU1 | India | 100.0 | 66.7 | 33 | 11 |
| 857 | NIAW SAN PAHTAWANG | Thailand | 100.0 | 100.0 | 27 | 15 |
| 3654 | JHONA | India | 100.0 | 14.3 | 27 | 3 |
| 4898 | GEB24 | India | 100.0 | 54.2 | 21 | 9 |
| 8948 | POKKALI | Sri Lanka | 100.0 | 50.0 | 22 | 7 |
| 9398 | OMIRT 39 | Hungary | 100.0 | 45.8 | 27 | 12 |
| 9810 | T 141 | India | 100.0 | 77.3 | 30 | 12 |
| 10829 | JHONA | Pakistan | 100.0 | 50.0 | 21 | 9 |
| 10869 | SML KAPURI | Surinam | 100.0 | 30.0 | 19 | 12 |
| 10870 | SML TEMERIN | Surinam | 100.0 | 35.7 | 13 | 8 |
| 15251 | MADAKALAPU SAMBA / 01-071 | Sri Lanka | 86.1 | 50.0 | 36 | 12 |
| 15314 | THAVALU / 03-003 | Sri Lanka | 100.0 | 42.9 | 30 | 7 |
| 15337 | POKURU SAMBA / 03-026 | Sri Lanka | 100.0 | 80.0 | 17 | 7 |
| 15430 | KALUKANDA / 04-047 | Sri Lanka | 100.0 | 71.4 | 10 | 9 |
| 15433 | VELI HANDIRAN / 04-050 | Sri Lanka | 100.0 | 68.2 | 17 | 5 |
| 19064 | TJEMPO RUNI / INDO NO. 5925 | Indonesia | 87.5 | 41.7 | 24 | 10 |
| 20829 | ARC 10288 | India | 100.0 | 68.8 | 31 | 8 |
| 21027 | ARC 10661 | India | 100.0 | 100.0 | 21 | 12 |
| 26449 | CHOTA BHAWALIA BRRI ACC. NO. 0100 | Bangladesh | 100.0 | 37.5 | 32 | 11 |
| 26774 | RAGU SAIL BRRI ACC. NO. 0414 | Bangladesh | 90.0 | 50.0 | 43 | 11 |
| 26802 | SUNGA WALA BRRI ACC.NO. 0515 | Bangladesh | 100.0 | 80.0 | 18 | 13 |
| 26814 | STG6511071 | United States | 71.4 | 50.0 | 29 | 14 |
| 26818 | RED RICE | Solomon Islands | 100.0 | 40.0 | 34 | 15 |
| 26830 | ADT29 | India | 100.0 | 50.0 | 36 | 15 |
| 26832 | ADT31 | India | 75.0 | 33.3 | 38 | 14 |
| 26907 | GOTTELU | India | 90.0 | 41.7 | 50 | 15 |
| 26913 | KALA-RATA 1-24 | India | 100.0 | 50.0 | 30 | 8 |
| 26936 | LANNAB 018 | Philippines | 100.0 | 8.3 | 37 | 12 |
| 26955 | CHIANUNG SEN 11 | Taiwan | 100.0 | 50.0 | 55 | 23 |
| 26956 | CHIANUNG SEN YU - 6 | Taiwan | 100.0 | 50.0 | 34 | 20 |
| 26962 | JUCHITAN A 73 | Mexico | 100.0 | 50.0 | 29 | 13 |
| 27485 | TOJUMA / INDO NO. 11-121 | Indonesia | 100.0 | 100.0 | 39 | 11 |
| 27590 | RAYADA 16-04 | Bangladesh | 100.0 | 91.7 | 32 | 8 |
| 28539 | JALADHI 2 | India | 100.0 | 83.3 | 45 | 10 |
| 28571 | TKM6 | India | 100.0 | 87.0 | 53 | 12 |
| 29858 | MONG CHIEM ACC. NO. C6924 | Vietnam | 100.0 | 70.0 | 40 | 4 |
| 29864 | NANG SAI ACC. NO. C6402 | Cambodia | 100.0 | 91.7 | 23 | 13 |
| 29873 | NEP VAN ACC. NO. C7045 | Vietnam | 90.0 | 42.9 | 30 | 4 |
| 29878 | PHEAR CHAM ACC. NO. C6239 | Cambodia | 100.0 | 75.0 | 36 | 5 |
| 29972 | KETTAU / H.K.A. 3 | Laos | 100.0 | 50.0 | 24 | 8 |
| 37167 | KHAMA CODE No 3B 010 | Bangladesh | 100.0 | 87.5 | 25 | 9 |
| 33382 | MEEGAUK | Myanmar | 94.0 | 86.4 | 25 | 19 |
| 33715 | SITPWA | Myanmar | 100.0 | 91.7 | 22 | 14 |
| 34152 | RIKUTO NORIN | Japan | 100.0 | 46.2 | 44 | 12 |
| 35185 | TKMG | India | 100.0 | 90.9 | 31 | 17 |
| 45858 | JALADHI 1 | India | 100.0 | 94.4 | 36 | 17 |
| 56628 | CHINOIS 6 | Senegal | 100.0 | 38.5 | 46 | 22 |
| 56717 | KALULU / ZS025 | Zambia | 100.0 | 50.0 | 60 | 17 |
| 56738 | CHINOIS TOS10505 | Guinea-Bissau | 100.0 | 92.1 | 56 | 15 |
| 57557 | BAYAR KUNING | Indonesia | 100.0 | 55.3 | 31 | 16 |
| 58911 | BARCHA BAHADUR | Nepal | 100.0 | 66.7 | 33 | 4 |
|  | SWARNA (check) | India | 100.0 | 34.0 | 23 | 12 |
|  | IR42 (check) | Philippines | 100.0 | 38.0 | 32 | 7 |
|  |  |  |  |  |  |  |

`

**Supplemental Table 2**. List of genotypes with best survival and their tiller number under severe stagnant flood conditions (20 cm flooding at 30 days after transplanting (DAT) followed by 40 cm at 37 DAT and 50-60 cm depth at 42 DAT) through maturity in 2007 wet season trial (from 223 entries); 2008 dry season (from 114 entries); 2008 wet season (from 112 entries) and their percentage survival. Data are from three replicates in each trial.

| Entry | Country of  Origin | Year & Season | % Survival | Tiller No. |
| --- | --- | --- | --- | --- |
| Lalmota | Bangladesh | 2007 WS | 89.0 | 17.0 |
| Vannan-Vellai | Sri Lanka | 2007 WS | 77.1 | 8.2 |
| IR80410-B-197-1 | Philippines | 2007 WS | 76.9 | 9.3 |
| BR23 | Bangladesh | 2007 WS | 75.0 | 11.3 |
| Jol Paira | Bangladesh | 2007 WS | 75.0 | 8.0 |
| Set Kumra | Bangladesh | 2007 WS | 74.7 | 9.8 |
| Dharikhachi | Bangladesh | 2007 WS | 69.6 | 7.8 |
| Patnai 23 | Bangladesh | 2007 WS | 68.9 | 8.8 |
| IR60608-2R-11-2-2-1 | Philippines | 2007 WS | 68.8 | 7.5 |
| IR80228H | Philippines | 2007 WS | 67.6 | 10.9 |
| Rajasail | Bangladesh | 2007 WS | 66.9 | 8.6 |
| Dorkumor | Bangladesh | 2007 WS | 65.7 | 12.0 |
| Nang Sai Acc.C6402 | Cambodia | 2007 WS | 64.8 | 7.8 |
| Tilakachari 826166 | India | 2007 WS | 64.3 | 5.7 |
| Madhumoti | Bangladesh | 2007 WS | 63.3 | 9.0 |
| Patnai 23 | Bangladesh | 2007 WS | 62.7 | 10.0 |
| IR64588-47-2-2B-9-2-2-3 | Philippines | 2007 WS | 62.5 | 8.8 |
| Bamonkhir | Bangladesh | 2007 WS | 61.1 | 12.0 |
| Ranisalute | Bangladesh | 2007 WS | 61.5 | 8.0 |
| IR79615-9-3-1-3 | Philippines | 2007 WS | 61.1 | 11.0 |
| IR81955H | Philippines | 2007 WS | 60.6 | 6.5 |
| Sirambeh Puti | Indonesia | 2007 WS | 60.2 | 9.0 |
| IR42 (check) |  | 2007 WS | 10.0 | 7.0 |
| Vannan-Vellai | Sri Lanka | 2008 DS | 66.4 | 4.8 |
| Sabita | India | 2008 DS | 61.3 | 3.3 |
| Ghigoj | Bangladesh | 2008 DS | 61.1 | 10.7 |
| Madhukar Code No. NC220 | India | 2008 DS | 60.2 | 6.1 |
| Jaladhi 1 | India | 2008 DS | 51.5 | 11.6 |
| Khama Code No.3B 010 | India | 2008 DS | 51.0 | 9.0 |
| Jaladhi 2 | India | 2008 DS | 50.0 | 6.6 |
| Katarangi | Bangladesh | 2008 DS | 41.5 | 8.0 |
| Dharikhachi | Bangladesh | 2008 DS | 40.0 | 10.6 |
| IR72667-16-B-B-3 | Philippines | 2008 DS | 40.0 | 9.0 |
| IR42 (check) |  | 2008 DS | 0.0 | 0.0 |
| Tilakachari 826166 | India | 2008 WS | 73.0 | 11.0 |
| Set Kumra | Bangladesh | 2008 WS | 71.7 | 11.0 |
| Chiknal | Bangladesh | 2008 WS | 70.0 | 10.0 |
| Ghigoj | Bangladesh | 2008 WS | 65.6 | 10.0 |
| Jol Paira | Bangladesh | 2008 WS | 64.9 | 13.0 |
| Patnai 23 | Bangladesh | 2008 WS | 62.1 | 12.0 |
| Rajasail | Bangladesh | 2008 WS | 57.0 | 11.3 |
| IR81958 | Philippines | 2008 WS | 52.0 | 9.3 |
| Mait Chal | Bangladesh | 2008 WS | 51.3 | 9.0 |
| Jaladhi 1 | India | 2008 WS | 51.0 | 12.0 |
| IR42 (check) |  | 2008 WS | 14.0 | 7.0 |

C. Sugars (Sub1 vs. Non-Sub1) D. Sugars (SF tolerant vs. SF intolerant)


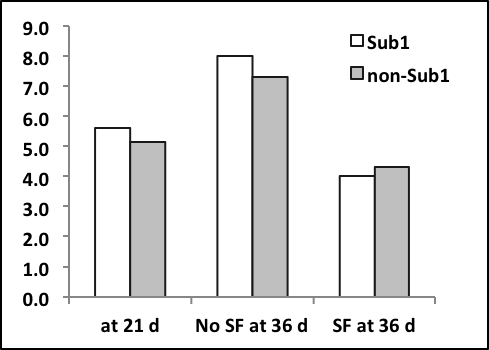


**n.s**.

**n.s**.

**n.s**.


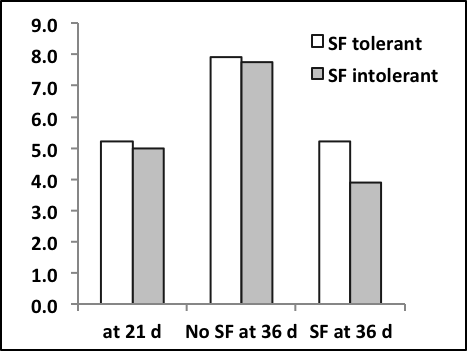


**n.s.**

**n.s.**

*****

A. Starch (Sub1 vs. Non-Sub1) B. Starch (SF tolerant vs. SF intolerant)


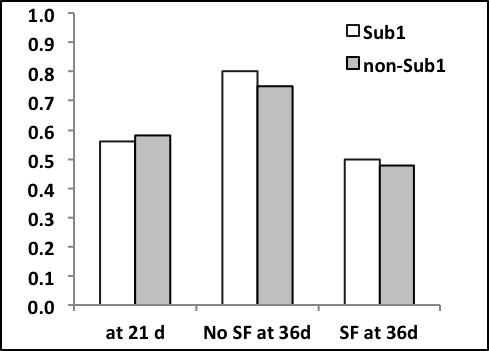


**n.s**.

**n.s**.

**n.s.**


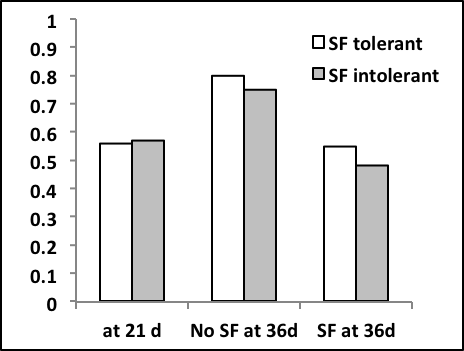


**n.s**.

**n.s**.

**n.s**.

**Supplementary Figure 1**. Comparison of stem starch (% of dry wt.) (A) between Sub1 and non-Sub1; (B) between SF tolerant and intolerant genotypes; and soluble sugars (C) between Sub1 and non-Sub1; (D) and between SF tolerant and intolerant genotypes. Data are means from 2009 and analyzed using Fishers’ test at P<0.001, n.s. indicate not significant; * significant at P<0.05. Data were collected before treatment (21 days after transplanting, DAT) and after 15 days under control and SF (36 DAT).
